# Nanomedicine in Bladder Cancer Therapy

**DOI:** 10.3390/ijms251910388

**Published:** 2024-09-26

**Authors:** Adrianna Winnicka, Joanna Brzeszczyńska, Joanna Saluk, Paulina Wigner-Jeziorska

**Affiliations:** 1 Department of General Biochemistry, Faculty of Biology and Environmental Protection, University of Lodz, 90-136 Lodz, Poland; adrianna.winnicka@edu.uni.lodz.pl (A.W.); joanna.brzeszczynska@biol.uni.lodz.pl (J.B.); joanna.saluk@biol.uni.lodz.pl (J.S.); 2 Department of Molecular Genetics, Faculty of Biology and Environmental Protection, University of Lodz, 90-136 Lodz, Poland

**Keywords:** bladder cancer, nanotechnology, nanoparticles, chitosan, cancer therapy

## Abstract

Bladder cancer (BC) is one of the most common malignant neoplasms of the genitourinary system. Traditional BC therapies include chemotherapy, targeted therapy, and immunotherapy. However, limitations such as lack of specificity, cytotoxicity, and multidrug resistance pose serious challenges to the benefits of BC therapies. Consequently, current studies focus on the search for new therapeutic solutions. In recent years, there has been a growing interest in using nanotechnology in the treatment of both non-invasive (NMIBC) and invasive bladder cancer (MIBC). Nanotechnology is based on the use of both organic molecules (chitosan, liposomes) and inorganic molecules (superparamagnetic iron oxide nanoparticles) as carriers of active substances. The main aim of such molecules is the targeted transport and prolonged retention of the drug in the target tissue, which increases the therapeutic efficacy of the active substance. This review discusses the numerous types of nanoparticles (including chitosan, polymeric nanoparticles, liposomes, and protein nanoparticles), targeting mechanisms, and approved nanotherapeutics with oncological implications in cancer treatment. We also present nanoformulation applications in phototherapy, gene therapy, and immunotherapy. Moreover, we summarise the current perspectives, advantages, and challenges in clinical translation.

## 1. Introduction

Bladder cancer (BC) is one of the most commonly recognised genitourinary system malignancies [1,2]. Statistically, the disease incidence in 2020 was estimated at 573,278 cases around the world with a mortality rate of 212,536 cases per year [3]. It is anticipated that the number of bladder cancer cases worldwide is expected to reach 991,000 in 2040. BC is the fourth most often occurring cancer in Poland among men, after prostate, colon, and lung tumours. However, epidemiologically, the prevalence of BC increases with age, affecting three times as many men as women [4]. Environmental factors contributing to the risk of bladder cancer include diets low in fruit and vegetables and urban lifestyle. Nevertheless, the most significant and common risk factor for bladder cancer is cigarette smoking, which is responsible for approximately 50% of cases. The main known carcinogens are beta-naphthylamine and polycyclic aromatic polycarbons, which promote inflammation and cause permanent genetic mutations [5]. The effect of alcohol consumption is, however, inconclusive in BC. Studies conducted among the Australian population did not show an increased risk of BC, while research among Japanese demonstrated a correlation between heavy alcohol consumption and BC [6]. Occupational carcinogen exposure, including aromatic amines, polycyclic aromatic hydrocarbons, and chlorinated hydrocarbons, is the second most frequent BC risk factor and is responsible for 5.7% of new BC cases [5,7]. Environmental factors, classified in the first group of carcinogens in IARC (International Agency for Research on Cancer), for example, high levels of arsenic, and in less-developed countries, air pollution (diesel and gasoline engine exhausts), have also been linked to BC development based on epidemiological studies [6,7]. Moreover, the risk of BC is higher among Caucasians compared to Asian and Black ethnic groups. This phenomenon may be explained by different smoking patterns and environmental exposures [7].

BC can be divided into NIMBC (non-muscle invasive bladder cancer) and MIBC (muscle-invasive bladder cancer), which have incidences of 75% and 25–30%, respectively [1,8,9]. Urothelial cancer is one of the most common histopathological types of BC (90%), and it is formerly known as transitional cell carcinoma (TCC) [8,9]. Unfortunately, BC is diagnosed too late due to its non-specific symptoms (e.g., urinary frequency, urgency, hesitancy, and dysuria), and it is often misdiagnosed as recurrent urinary tract infections [1,2,9]. Moreover, BC is characterised by the incidence of recurrence. After five years, the risk of NMIBC recurrence may reach 78%, and the rate of progression to MIBC is 45%. Therefore, accurate diagnosis and effective treatment are crucial. For patients with suspected BC, the first course of action is to undergo cystoscopy and urine cytology, which is considered to be a diagnostic “gold standard” [10,11]. MRI (magnetic resonance imaging) and CT (computerised tomography) imaging are used to determine the stage of disease advancement. After diagnosis, it is of key importance to choose an adequate treatment method according to the stage and type of BC [11]. One of the challenges that current BC research faces is the lack of specificity toward tumoural transformed cells that, as a consequence, cause cytotoxicity in healthy tissues. Furthermore, the development of drug resistance in BC pathogenesis increases the difficulty in clinical cancer management. Unfortunately, the chemotherapeutics in use are characterised by low solubility and weak bioavailability [12]. Due to the associated difficulties in BC therapy and poor patient clinical outcomes, it is necessary to develop new treatment strategies tailored to the individual needs of BC patients. Nanoparticles (NPs) seem to have such potential [13]. Nanoparticles show promise for use in cancer treatments, including BC, thanks to their unique physicochemical properties: small size effect, large specific surface area, high reactivity, and also quantum effect [14,15]. Designed nanoparticles through linking with therapeutic agents or through encapsulation can be delivered. This process can occur passively or actively at the tumour location. The passive targeting of NPs takes place because of the unique structure of cancer tissue. The atypical and porous blood vessels of a tumour result in the process of enhanced permeation and retention, which leads to the gathering of NPs. However, this transport has its drawbacks, as it can lead to incorrect targets and, as a consequence, a dispersion of the drug effect. Active targeting is another mechanism, which results in the binding of active molecules (peptides, antibodies, amino acids) on the surface of NPs. NPs attach to specific cancer cell receptors, which results in endocytosis and drug release [16,17].

Currently, nanoparticles are divided into organic and inorganic types. In the organic group, liposomal and polymeric nanoparticles are included, and in the inorganic group, e.g., magnetic nanoparticles, gold nanoparticles and mesoporous nanoparticles are included.

The objective of this article is to present the current state of knowledge on nanoparticle use in personalised BC therapy based on a review of the available literature.

## 2. Conventional BC Therapy

Depending on the grade and stage of a tumour (based on the Tumour–Node–Metastasis classification system, 1973), general health, and patient preferences, different types of therapies are available for BC [18]. BC stage classification is presented in Table 1.

As already mentioned, we distinguish the NMIBC and MIBC forms. The preferred conventional treatment for NMIBC is transurethral resection of bladder tumour (TURBT) [19,20]. This method has two main purposes, which are to resect all visible tumours and clinically stage the tumour. TURBT can be performed either under spinal or general anaesthesia and involves the insertion of a resectoscope through the urethra [21]. The research indicates that after the first surgery, 50–70% of tumours recur, while 10–30% progress further within 5 years. To notice the early recurrences after a successful TURBT, regular cystoscopy is necessary. This, however, is associated with the downgrade of general life quality and high economic costs [22]. Some patients must undergo re-TURBT within 4–6 weeks after the first surgery when there is incomplete resection or a lack of detrusor muscle in the pathologic specimen [4]. For low-risk and intermediate-risk NMIBC, TURBT can be followed by single-dose intravesical chemotherapy (SI) based on mitomycin C, gemcitabine or less common alternatives epirubicin and doxorubicin. This approach can prevent the implantation of residual cancer cells after the TURBT procedure and reduce the risk of recurrence from 12 to 14%. Among the most common side effects of SI is urinary irritation, although fat necrosis, fibrosis, urinary leakage, and fistula formation can rarely occur [23]. Immunotherapy with Bacillus Calmette Guerin (BCG), a live attenuated strain of *Mycobacterium bovis*, is the oldest remedy applied in immunotherapy for BC treatment since it was first used by the Morales team in 1976 on nine patients with superficial bladder cancer (TCC). Nowadays, it is the recommended treatment for high-risk NMIBC and it is optional for intermediate-risk NMIBC. This approach involves the installation of BCG into the bladder for 90–120 min. The procedure is carried out once a week for 6 weeks. In the case of good patient tolerability and a good clinical response, maintenance therapy is maintained for 1 year (intermediate-risk group) and up to 3 years (high-risk group) [4]. The BCG vaccine works as an immunomodulating agent, which enhances the immunological response [24] and has similar side effects as intravesical chemotherapy [23]. Its mechanism covers several stages: the attachment of BCG to the urothelium, which is followed by the internalisation of BCG into resident immune cells, normal cells, and tumour urothelial cells; and finally the stimulation of the immune system to produce higher concentrations of cytokine and interferon-gamma. However, for approximately half the patients, the therapy results in failure [24]. It is estimated that the risk of recurrence within 2 years is 60%, while the risk of progression to the advanced stage is 25% [25,26,27,28]. Usually, after BCG failure, the chance of responding to another cycle of BCG is unlikely. In this case, for patients with intermediate-risk or high-risk disease [23,29], AUA guidelines recommend several options such as radical cystectomy, intravesical therapies or clinical trial enrolment [30]. Intravesical therapies can be conducted by using doxorubicin, gemcitabine, docetaxel, or valrubicin [23]. Valrubicin is effective in 20% of all cases and is the only FDA (Food and Drug Administration)-approved drug for patients with carcinoma in situ (CIS) [25]. For patients with BCG-unresponsive NMIBC, there is also an option of systemic immunotherapy with pembrolizumab. Due to the occurrence of radiotherapy resistance, it is currently not used in NMIBC [23].

Regarding MIBC, the standard treatment is radical cystectomy (RC) with neoadjuvant platinum-based chemotherapy. RC is considered one of the most challenging operations in the field of urology, and it can be conducted in two ways: robot-assisted RC (RARC) and open RC (ORC). This procedure involves the removal of the bladder, adjacent organs (e.g., prostate, seminal vesicles, female reproductive organs), and regional lymph nodes. The EAU (European Association of Urology) guidelines recommend performing RC within 3 months of diagnosis. However, this method does not give satisfying results for all BC patients, which is a significant issue. Moreover, the RC is characterised by a high percentage of complications, which are estimated between 30 and 70% [31]. The five-year survival rate after RC without prior neoadjuvant chemotherapy is only 50%. Therefore, neoadjuvant chemotherapy has become a major step in reducing the risk of recurrence and improving the overall survival of MIBC patients [20]. Because of the high sensitivity of advanced BC to platinum-based combination chemotherapy [32], NAC (neoadjuvant chemotherapy) uses accelerated or dose-dense methotrexate, vinblastine, doxorubicin, and cisplatin (MVAC, ddMVAC) or gemcitabine and cisplatin (GC) [4]. The main goal of pre-chemotherapy is to reduce the size of the tumour and to treat micrometastases [33]. A meta-analysis involving 976 patients revealed a significant 16% reduction in the risk of death with the use of cisplatin, methotrexate, and vinblastine (CMV). Furthermore, this change resulted in an increase in the 10-year survival rate from 30% to 36% [34] For some patients who cannot undergo RC, bladder-conserving treatments are available. Trimodality therapy (TMT) is among those treatments. It consists of TURBT followed by simultaneous chemotherapy and radiotherapy (RT). For chemotherapy, radiosensitising agents are used such as cisplatin, mitomycin C (MMC)/5-fluorouracil (5-FU), gemcitabine, carbogen-nicotinamide and panitumumab (monoclonal antibody). Based on current protocols, RT regimens include an external beam (up to 40 Gy) to the bladder and pelvic lymph nodes, next increasing the dose to 54 Gy for the whole bladder, and finally targeting the tumour bed with a total dose of 64–65 Gy. TMT is in general well tolerated, but 10–36% of patients experienced acute toxicity [35]. Approximately 70% of patients showed clinical response (CR) to TMT. RTOG trials indicated that 5-year OS and disease-specific survival were 57% and 71%, respectively. It is noteworthy that after TMT, 80% of patients will preserve an intact bladder, while 75% will have a functioning normal bladder [36].

Another form of BC treatment is phototherapy, including photothermal and photodynamic therapies (PDT), using light of particular wavelength to destroy the cancer tumours. PDT is a modern method of treatment of many kinds of malignant tumours (e.g., of lungs and digestive system) and is an innovative technique in oncological urology. PDT relies on the administration of a photosensitising agent (PS) to the tumour location, which is then activated by light energy of appropriate wavelength [37]. As a result, oxygen is transformed into its reactive forms (ROS) and leads to apoptosis and necrosis of the cancer cells [9]. Kwiatkowski et al. (2018) in their research used hematoporphyrin derivative as a photosensitiser to determine the effectiveness of PDT in therapy for superficial transitional cell carcinoma of the bladder. A decisive element of PDT therapy is the choice of an appropriate photosensitiser [38]. An example of such a sensitiser used in PDT is photofrin, which is characterised by lipophilicity. Lipophilic photosensitisers penetrate mitochondria, damaging them in the process. Thus, cytochrome C is released from the mitochondria, leading to the activation of the intrinsic pathway of apoptosis and therefore death of the cell [25]. There are three generations of PS used in PDT: hematoporphyrin and its derivatives (I generation), 5-aminolevulinic acid (II generation), and photosensitisers based on nanoparticles (III generation) [39]. Because of the limited ability to penetrate [40], phototoxic reactions occur only within the cancerous tissues only if the correct photosensitiser is distributed. Thanks to that, the targeted death of only cancer cells is possible. However, shallow penetration through the pathological tissue and the toxicity toward normal cells result in certain limitations for clinical therapy [9].

## 3. Nanoparticles in BC Therapy

Intravesical drug delivery is an important treatment for BC; however, due to the anatomical structure of the bladder, there exist some limitations. The bladder permeability barrier (BPB) is an anatomical barrier to the diffusion of anticancer drugs from vessels, which for this reason requires several administrations of the drug and, hence, it is associated with complications. Therefore, it is crucial to find a solution that would increase the contact time of the drug with the changed tissue [41,42]. Nanoparticles have revolutionised the field of medicine by significantly improving the efficacy of therapeutic interventions. One of the most significant advantages of nanoparticles is their high surface area-to-volume ratio. This property enables nanoparticles to interact more efficiently with their surroundings, making them invaluable in catalysis, drug delivery, and sensor applications. The increased surface area allows for greater exposure to reactants, leading to enhanced reactivity and efficiency in chemical reactions. In cancer treatment, for example, nanoparticles can selectively accumulate in tumour tissues via the enhanced permeability and retention (EPR) effect. This selective targeting allows for higher drug concentrations at the tumour site while minimising systemic toxicity. Mucoadhesive molecules, due to their hydrophilic nature, form a large number of hydrogen bonds with mucin of the urothelium mucosa, which in turn allows the preparation to be retained at the site of action for a long time [39]. Mucoadhesive molecules include chitosan or synthetic polymers. If these preparations are used in BC therapy, they should meet three basic criteria: adhesion to the bladder wall; no effect on urine flow and kidney function; and adhesion to the altered tissue after urination [43].

Currently, several nanosystems for the treatment of BC are in clinical trials. One of the clinical trials is a study (NCT0276996) determining the overall response rate of EP0057 in combination with Olapirib among BC/castrate-resistant prostate cancer (mCRPC) patients. EP0057 is nanoparticle–drug conjugate (NDC) of a cyclodextrin-based polymer backbone plus camptothecin, which is a topoisomerase-1 inhibitor. In contrast, a clinical trial (NCT03382340) is investigating the use of Imx-110 (curcumin/doxorubicin-encapsulating nanoparticle) in the treatment of solid tumours. Another promising clinical trial (NCT05519241), which is still in the recruiting phase, evaluates PLZ4-coated paclitaxel-loaded micelles (PPM) in BCG-unresponsive NMIBC treatment (Table 2) [43].

### 3.1. Chitosan Nanoparticles

Chitosan is a natural linear cationic polysaccharide resulting from the alkalic hydrolysis of chitin—the natural component of the cellar walls of fungi, some fish and invertebrate structures. Due to its biodegradability, non-toxicity and biocompatibility, chitosan has potential clinical use [44,45,46,47].

Until now, much research has been conducted on chitosan and its derivatives nanocarriers as platforms for synergic drug delivery in BC treatment (Table 3). Şenyiğit et al. (2015) have used the chitosan–thioglycolic acid (chitosan-TGA) complex as a delivery system for gemcitabine hydrochloride (Gem-HCL). In vitro analyses showed that the covalent cross-linking used to combine the drug with the chitosan-TGA complex significantly reduces the release rate. Moreover, histopathological tests confirmed that chitosan nanoparticles loaded with Gem-HLC do not damage the bladder mucosa; therefore, they can be considered safe [48]. Liu et al. (2018) through a simple co-assembly method managed to create paclitaxel/chitosan (PTX/CS) nanosuspensions (NSs), which are marked with high drug-loading efficiency [49]. Paclitaxel is one of the most popular first-line cytotoxic agents to treat the vast majority of cancers, like BC [50,51]. To verify the effectiveness of the drug (PTX release, cell uptake, and cytotoxicity), in vitro tests have been conducted, while the reference time in the bladder and antitumour effect was evaluated in vivo using an animal model. Drug release studies showed a slow drug release profile from PTX/CS. MTT (3-(4,5-dimethylthiazol-2-yl)-2,5-diphenyl-2H-tetrazolium bromide) assay and CLSM (confocal laser scanning microscopy) indicated good cellular uptake and efficiency in killing the cancer cells. An in vivo study on a BC mice model established an antitumour effect. In mice from the control group, faster tumour growth was observed compared to groups treated with PTX/CS blends and PTX/CS NSs. Moreover, the PTX/CS NSs group had better treatment results than the PTX/CS blends group. After having conducted the autopsy, the size of the tumour was evaluated, which was significantly larger in the control group. Evaluation of toxicity of the PTX/CS NSs showed limited side effects. PTX/CS NSs can be considered promising tools in BC therapy because of high PTX loading efficiency, sustained PTX release, positively charged surface, good mucin adhesion, low toxicity, and increased antitumour efficacy [49].

Another study by Xu et al. (2020) focused on using BC tumour-specific nanomedicine [52]. This research involved the use of hydrophobically modified chitosan to deliver a prodrug of gambogic acid which will be activated in the presence of a high level of ROS. To obtain high encapsulation efficiency of hydrophobic payloads, chitosan was modified by adding a benzyl group to hydrophobic moieties. The in vitro (MB49; mouse urothelial carcinoma cell line and NIH-3 cells; mouse embryonic fibroblast cells) and in vivo studies (C57BL/6 J mice model) were conducted to establish the cytotoxicity, detection of apoptosis, cell cycle arrest, cellular uptake, mucoadhesiveness and permeability of free drugs and nanomedicines in the bladder. The internalisation of nanoparticles by MB49 cells was monitored using CLSM. Incubation with a lipophilic membrane stain (Dil) confirmed the great mucoadhesiveness of modified chitosan. Because of the electrostatic interaction between positively charged chitosan and the membrane, enhanced fluorescence was observed at 9 h after 3 h incubation with DiI. The cytotoxicity assay showed the selectivity of prodrug nanomedicine toward tumours as well as the ability to efficiently transform into the parent GA inside cancer cells. MB49 cells treated with the free drug GA, GB, and nanoformulations showed a concentration-dependent inhibition of proliferation. Staining with propidium iodide (PI) and Annexin-V FITC revealed that free GA contributed to 34.7% of early apoptotic cells and 33.3% of late apoptotic cells. The level of free GB was up to 33.6% and 33.7%, respectively. Weaker proapoptotic ability was observed for the nanoformulations of both GA and GB. Mucoadhesiveness was confirmed by the small animal living imaging system. Strong fluorescence was observed in the bladders of mice that were treated with encapsulated GB in comparison with the bladders of free drug mice. The fluorescence intensity of the free drug decreased rapidly after instillation, but the excretion of the nanodrug exhibited a much slower velocity. This improved the mucoadhesiveness properties of encapsulated GB in comparison with the free drug. The permeability of free drug and positively charged nanomedicine, the penetration depth of free DiI, and DiI labelled nanoformulations were detected by CLSM. The penetration of free Dil was confined only to the mucosa. In contrast, DiI nanoformulas showed a much stronger ability to penetrate the mucosa. To determine the antitumour properties, the C57BL/6 J mice had an intravesical injection of MB49 cells after acid-induced urothelium damage. The nanoformulation of GB showed a significant inhibition of tumour growth in the BC model and no toxicity to a normal urothelium [52,53].

**Table 3:** Chitosan in BC drug delivery.

Nanocarrier	Drug	Amount of Nanoparticle Formulations	Results	Study Type	Days of Study	Ref.
Chitosan (CS)/poly-epsilon-caprolactone coated with chitosan (CS-PCL)	Mitomycin C (MMC)	100 μL	Satisfactory drug loading and release profiles, antitumour activity, good cellular interaction.	In vitro (MB49 mouse urinary bladder carcinoma cell line)	-	[54]
Glycol chitosan (CDDP-HGCs)	Cisplatin (CP)	10 mg/kg	High drug loading efficiency, sustained release of CP, ability to tumour targeting, antitumour activity, low toxicity.	In vivo (tumour-bearing mice)/in vitro (squamous cell carcinoma (SCC7) cells, A549 human lung cancer cells)	25 days	[55]
Hydrotropic oligomer-glycol chitosan (HO-GC)	Paclitaxel	5 mg/kg	High drug loading efficiency, enhanced permeation and retention effect and low toxicity.	In vivo (athymic nude mice)/In vitro (MDA-MB231 human breast cancer cells)	3 days	[56]
Chitosan (CS), chitosan/polyethylene glycol (CS/PEG)	Indole-3-carbinol (I3C)	0.0–1000 μM	Reduction in cancer cell viability and high drug release profiles.	In vitro (human bladder cancer cell line (T24)	-	[57]

### 3.2. Polymeric Nanoparticles

Polymeric nanoparticles (PNPs) are submicron-sized colloidal systems, which can be obtained from natural or synthetic polymers [57,58,59,60]. Among the macromolecules creating PNPs, we can distinguish “poly (lactide-coglycolide)”, “poly (lactic acid)”, “poly (ε-caprolactone)”, “chitosan”, and “poly (alkyl cyanoacrylates)” [61]. In the production of PNPs, there is a possibility of scale-up, even under Good Manufacturing Practices (GMPs). This is in contrast to the production of other nanocarriers, such as micelles, liposomes, or organic nanosystems [62]. PNPs have many advantages when it comes to the active substances delivery system. Among them, there is high encapsulation efficiency, high intracellular uptake, high stability of active substance encapsulation, biocompatibility with tissues and cells, and biodegradability when they are composed of biopolymers. Those features are crucial for their use in biomedicine. Biologically active molecules can be absorbed on the surface or from within the nanoparticles [63,64,65]. Encapsulated drugs can be released in a controlled way through diffusion with a polymer matrix, chemical degradation of the particle or both of these processes [66,67]. Biodegradable polymers are bound to decay into monomers, which then are metabolised and removed from the organism. By modifying the side chain of the polymer, the pace of the polymer’s decay and the release of the drug can be impacted [68]. PNPs also have possible disadvantages that must be taken into consideration, such as no biodegradability, frangibleness, high manufacturing costs, and toxic solvent residuals [57,69].

Kates et al. (2017) studied nanoparticle-based CDDP (cisplatin) for non-toxic and efficacious IDD. CDDP NPs were based on biocompatible poly(l-aspartic acid sodium salt; PAA) or methoxy-poly(ethylene glycol)-block-PAA (PEG–PAA) with or without different densities of PEG (high or low). The conducted in vitro study on superficial bladder cancer cell lines (RT4) and invasive cell lines (J82 and 5673) indicated higher values of IC_50_ for all types of CDDP NP compared to free CP. After the intravesical administration of NPs, fewer side effects were observed. Furthermore, the hyperplasia of bladder tissue and increase in bladder weight were not noticed, whereas these findings appeared after free CP administration. An interesting result of the research was the fact that PAA-CDDP NPs increased the concentration of CP in the bladder tissue, while the PEG nanoparticles did not result in such an effect. Ultimately, the PAA-CDDP NPs showed high antiproliferative activity in a rat’s model NMIBC [70]. Huang et al. (2012) used the connection of polymer nanoparticles and superparamagnetic iron oxide nanoparticles (SPIONs) as a CP delivery system in BC therapy. The amphiphilic poly(ε-caprolactone)-b-poly(propargyl methacrylate-click-mercapto succinic acid-co-poly(ethylene glycol) methyl ether methacrylate)(PCL-b-P(PMA-click-MSA-co-PEGMA) nanoparticle synthesis occurred in three stages: ring-opening polymerisation, addition−fragmentation chain transfer (RAFT) polymerisation, and thione “click” reaction. After the reception of SPION nanoparticles, they were “loaded” to the PCLs core, while with the use of di-carboxylic groups appearing on the nanostructure’s surface, the CP was linked. The Pt−Fe−PN received that way had mucoadhesive and supermagnetic properties. The mucoadhesion of Pt-Fe-PN has been confirmed by the mucin-particle method. Cisplatin release was investigated by using the dialysis method. During the first four hours, 30% of CP from artificial urine was released, and after 4 days, it reached 41%. Moreover, the increase in the temperature increased the velocity of the drug’s release. The cytotoxicity evaluation in vitro of Pt−Fe−PNs on UMUC3 bladder (human urothelial carcinoma cell line) showed high antitumour activity toward the bladder tumour [71].

Docetaxel (DTX) is a half-synthetic taxane and a side-chain analogue of paclitaxel. It is acquired from the European yew (*Taxus baccata*). Docetaxel has a different structural configuration than paclitaxel and also possesses better solubility. It shows a high connection to β-tubulin in microtubules. This is why the main action of the taxane is to stop the cell cycle in the G2/M phase, which, as a consequence, leads to the apoptosis of the cell. Although paclitaxel and docetaxel mechanisms of action are similar to each other, the antitumour activity is different [72]. Docetaxel (DTX) nanoformulation for intravesical bladder cancer therapy was investigated by Mugabe et al. (2011). Mucoadhesive nanoformulations (HPG-C8/10-MePEG) were established by modifying hyperbranched polyglycerols (HPGs) with methoxy-polyethylene glycol (MePEG). To enhance mucoadhesive properties, the derivatisation of outer surface hydroxyl groups (HPGC8/10-MePEG-NH2) was conducted. All developed DTX nanoformulations (HPGC8/10-MePEG-NH2, HPG-C8/10-MePEG) and commercial formulations of taxotere indicated cytotoxicity against the KU7-luc cell line, low-grade (RT4, MGHU3) and high-grade (UMUC3) human urothelial carcinoma cell lines. However, the highest inhibition of tumour growth in KU7-luc was observed with DTX-loaded HPG-C8/10-MePEG-NH2. A single intravesical instillation of DTX-loaded HPGC8/10-MePEG-NH2 to a mice model caused an 80% inhibition when compared to the PBS control group. The nanoformulation without enhanced mucoadhesive properties (DTX-loaded HPG-C8/10-MePEG) inhibited only 54%. Interestingly, the commercial formulation of Taxotere was incapable of inhibiting tumour growth in an orthotopic xenograft model. Most importantly, DTX-loaded HPG-C8/10-MePEG-NH2 had much better accumulation in bladder tissue in comparison with taxotere or DTX-loaded HPG-C8/10- MePEG and also increased drug uptake in mouse bladder tissues [73].

To increase the mucoadhesive capacity of the nanocarrier and simultaneously the penetration of the chemotherapy drug, Guo et al. (2020) developed a positively charged nanogel of oligoarginine-poly(ethylene glycol)–poly(L-phenylalanineco-L-cystine) (R9-PEG–P(LP-co-LC)). Non-specific interactions between negatively charged mucosa membranes and the PEG chain were the result of enhanced mucoadhesion. As a model medicine, 10-hydroxycamptothecin (HCPT) was used. Through facile diffusion, it was encapsulated to the core of R9-PEG-P(LP-co-LC). R9NG/HCPT acquired this way shows higher cytotoxicity against human BC 5637 cells than free HCPT and NG/HCPT. In the case of R9NG/HCPT, a greater percentage of apoptotic cells was observed, which was about 22.5%. After the intravesical administration of HCPT nanoformulations to the rats, the fluorescence intensity of R9NG/HCPT was stronger than that of free HCPT, and it also remained relatively constant for an extended period, which indicates high mucoadhesion toward the urothelial surface. Over time, R9NG/HCPT could penetrate the full thickness of the bladder. The research conducted on murine and rat orthotopic BC models showed high antitumour activity. Based on the analysis of the weight loss and the survivability of the rats subjected to the R9NG/HCPT, low systematic toxicity was determined. Given the results acquired through the experiment, R9NG/HCPT has potential for clinical applications [74].

Table 4 summarises the studies presented in the section ‘Polymeric nanoparticles’.

### 3.3. Lipid-Based Nanoparticles

Lipid-based nanoparticles (Lipid-NPs) is a nanoparticles group including liposomes, lipid nanoparticles (LNPs), nanoemulsions (NEs), solid lipid nanoparticles (SLNs), nanostructured lipid carriers (NLCs), extracellular vesicles (EVs), and lipid polymer hybrid nanoparticles (LPHNPs) [75].

Liposomes, which are lipid-based structures, are also a prototype of lipid-NPs. They create spherical follicular systems with a variety of sizes. They can be composed of natural or synthetic phospholipids, which create a double lipid layer, surrounding an aqueous core [76]. Because it resembles structures responsible for creating cell membranes, liposomes are considered safe, with high biocompatibility, degradability, and low toxicity [75,77].

Amphiphilic character ensures the ability to transfer both hydrophobic and hydrophilic drugs [75,76,77,78,79]. The liposomes were first described in 1961 by Bangham [80], and they are currently known as one of the best medicine carriers [81]. They have various applications such as protecting drugs from in vivo degradation, controlling drug release, targeted drug delivery, modulating biodistribution and enhancement of solubility, and bioavailability [77]. Moreover, by manipulating the percentage of components, the surface load of lipids, as well as the presence of surface ligands, it is possible to identify the physicochemical properties and biological effectiveness of liposomes [75]. The above-mentioned modifications prevent short circulation time in human body fluids, instability in vivo and lack of targeting selectivity, which are the main flaws of unmodified liposomal carriers [82,83,84].

Kaldybekov et al. (2018) designed a drug delivery system, based on the use of a mucoadhesive carrier, applied in BC therapy—maleimide-functionalised PEGylated liposomes [85]. The encapsulation efficacy and loading capacity of lipid nanocarriers (conventional, PEG-Mal and PEGylated liposomes) were carried out using NaFI as a model drug. The encapsulation efficiency (EE%) of conventional liposomes was 53 ± 6% and had the highest efficacy among other liposomes. PEG-Mal and PEGylated had % EE values of 25 ± 2% and 27 ± 2%, respectively. The flow-through method with fluorescent detection showed a high mucoadhesive activity of PEG-Mal. 32% of PEG-Mal, and 18% of conventional liposomes stayed attached to the bladder mucosa after treatment with AU (artificial urine). Therefore, the increased mucoadhesive capability of PEG-Mal resulted from creating covalent bonds between maleimide groups and thiols groups, which occur on the surface of the mucin layer. Conducted fluorescence microscopy demonstrated greater penetration abilities of PEG liposomes than PEG-Mal, which only confirmed reports from other sources that pegylation leads to a lowering of the number of interactions with biological tissues, resulting in deeper penetration. In comparison with conventional liposomes, the in vitro study conducted with a dialysis method indicated a longer profile of PEG liposomes and PEG-Mal release. This can provide better drug effectiveness and longer preservation of the therapeutic dose after the administration [85].

Curcumin is a derivative of polyphenol, which is isolated from turmeric rhizome. In conducted in vitro and in vivo studies, its high antitumour effectiveness has been noticed. Curcumin also shows anti-inflammatory, anti-oxidant, anti-bacterial anti-diabetic, anti-fungal, anti-prolific and hepatoprotective properties. For these reasons, it has many therapeutic uses. Because of curcumin’s hydrophobic nature, which makes it more difficult to penetrate through cellar membranes, there has been increased interest in nano-based formulations of polyphenolic curcumin in recent times [86]. Gholami et al. (2023) designed liposome formulations based on soybean phosphatidylcholine (SPC) and hydrogenated SPC (HSPC) for the delivery of anticancer agent curcumin to human cancer cells [87]. In vitro studies on the cellular line of the bladder cancer HT89 and a normal cellular line of the fibroblasts, L929, indicated the no-stop release of curcumin from the liposomal nanoparticles. The SPC and HSPC specimens have also increased the cellular uptake and curcumin’s cytotoxicity in comparison to cancer cells. It was also shown that the nanoliposomal formulation of curcumin induces apoptosis and DNA damage, which results in a decreased lifespan of cancer cells [87]. Curcumin nanoformulation was also studied in a double-blind placebo-controlled trial by Sandoughdaran et al. (2021) [88]. This study was designed to determine the effect of nanocurcumin supplementation among MIBC patients undergoing chemotherapy. The study included 26 patients who were randomly assigned to two groups. The first group received 180 mg/day of nanocurcumin (Sina Curcumin), and the second group received a placebo. To assess the complete clinical response, the first follow-up was conducted four weeks after the end of the treatment. The second follow-up aimed to determine chemotherapy-induced nephrotoxicity, hematologic nadirs and toxicities between the two groups. Results showed that nanocurcumin was well tolerated, and the clinical response was estimated at 30.8% and 50% for the placebo and nanocurcumin groups, respectively. Unfortunately, there was no statistically significant difference between the groups (*p* = 0.417). Currently, no other data are available regarding the next phase of this study [88].

Table 5 summarises the studies presented in the section ‘Lipid-based nanoparticles’.

### 3.4. Protein Nanoparticles

Protein nanoparticles are built from biological particles of animal or plant origin, which makes them highly biocompatible, non-toxic, biodegradable and non-antigenic [61,89]. Thanks to the presence of multiple amino and carboxylic acid groups on the nanoparticle’s surface, a specific orientation toward cells or ligands is possible. The protein nanoparticles are capable of transferring both minor and major particles. Among the studied proteins which can be used as a tool to create nanoparticles are albumin, gelatin, gliadin and legumin [61]. Gelatin is the first material used to form nanoparticles. It is a natural soluble polymer, which is received through collagen hydrolysis. Because of its low mechanical durability and fast-paced decay, modification of the particles through cross-linking is necessary, e.g., with glutaraldehyde [90,91]. Albumin is a globular protein, which occurs in the circulatory system. Throughout the years, it became a powerful macromolecular carrier in medicine. Protein nanoparticles have been used to deliver numerous types of hydrophilic and hydrophobic drugs [91].

Lu et al. (2011) developed gelatin nanoparticles to deliver the hydrophobic drug paclitaxel (PTX) in the BC treatment [92]. Nanoparticles containing paclitaxel were prepared with the desolvation method. In vivo studies were conducted on tumour-bearing dogs (various breads) and free-tumour beagle dogs by using a urethral catheter to administer the designed nanoformulation. After 2 h of incubation, the urine sample was collected. To confirm drug concentration, tissue samples (urothelium and lamina propria) were harvested at different times (4, 8, 24, 72, 168 h). The study results showed that in dogs treated with intravesical PNP, paclitaxel was measurable in the plasma up to 8 h after 2 h treatment. Drug concentration in tumours was 40 μg/g, and a 360-fold higher average of total PTX was observed in bladder tumours compared to tumour-free dogs. Approximately only 1% of the intravesical administrated PNP dose was absorbed; hence, this treatment can be considered safe. The average drug concentration in collected tissues at 4 h was 13 times higher than free-PTX in the urine. Moreover, this study showed that gelatin nanoparticles are a better form of drug delivery than Cremophor micelle formulation. The concentration of PTX from gelatin nanoformulation was 5.4 times higher than that with a micellar formulation. Even though results showed major findings and indicated that PNPs have all the required properties for IDD, clinical studies are necessary [92].

CD47 is an innate immune checkpoint, which mainly aims to mediate neutrophil migration and T-cell co-stimulation. The high expression of the mentioned surface protein is observed in NMIBC as well as in MIBC [93]. For this reason, it seems to be a good therapeutic target. Mullapudi et al. (2022) designed a human albumin nanocarrier (txCD47-HNP) for the delivery of gemcitabine elucidates (GEMs), which targeted bladder cancer cells with a high expression of receptor 47 [94]. The study was conducted in vitro as well as in vivo. In vitro results showed that the IC_50_ of GEM nanoformulation was 10 times slower than that of free GEM. In vivo studies indicated a reduced tumour growth of mouse orthotopic BC model after treatment with GEM loaded in txCD47-HNP. After labelling with fluorescent mark txCD47-HNP, more than 83% of tumour cells in BC patients exhibited fluorescence. Hence, txCD47-HNP can potentially serve as targeted drug delivery in NMIBC and also as an alternative to urine cytology [94].

Table 6 summarises the studies presented in the section ‘Protein nanoparticles’.

## 4. Application of Nanoformulations in Phototherapy

In recent years, there has been an increasing interest in the use of nanotechnology in phototherapy. Photo-mediated therapies include photodynamic therapy (PDT) and photothermal therapy (PTT), whose mechanism of action is based on cell damage through the induction of the production of reactive oxygen species or thermal damage. In the case of BC, PDT is particularly used. Despite its high therapeutic potential, PDT has several limitations that can be overcome by the combined use of PDT and nanoparticles. Combining nanomaterials and photosensitisers may prevent side effects as well as increase the effectiveness of PDT. The therapeutic effect of PDT is attenuated by the hypoxic tumour environment, which reduces the efficiency of ROS production at the target site. The solution to this problem is to develop nanoparticles conjugated with molecules such as catalase, which can generate oxygen, or haemoglobin and perfluorocarbon, which serve as an oxygen carrier. Therefore, the incorporation of these molecules into nanoparticles can improve the effectiveness of PDT. The effectiveness of PDT is also affected by the poor solubility of photosensitising drugs, which interferes with the proper distribution of the drug to the target tissue. This limitation can be minimised by using nanoparticle systems that increase drug solubility and cellular uptake. In addition, the encapsulation of photosensitising drugs ensures targeted transport to cancer cells, reducing their toxic effect on normal tissues [38,95].

Many studies have been conducted to date to minimise the limitations of PDT in BC therapy. Yan et al. (2013) developed 5-ALA (5-aminolevulinic acid)-loaded nanoparticles and determined their phototoxic potential on T24 bladder cancer cells in vitro [96]. The foundation of a nanoparticle was caprolactone-polyethylene glycol-lactide, which was obtained using a ring-opening copolymerisation method. First, 5-aminolevulinic acid was loaded into the nanoparticle by using the nanoprecipitation method. MTT assay showed enhanced cytotoxicity of 5-ALA-loaded nanoparticles compared to 5-ALA-free drug. Also, a better inhibition effect (more than two times) of 5-ALA nanoformulations compared with free-5-ALA was determined. These findings indicated that nanomodified 5-ALA enhanced the effectiveness of photodynamic therapy [96].

**Figure 1:**
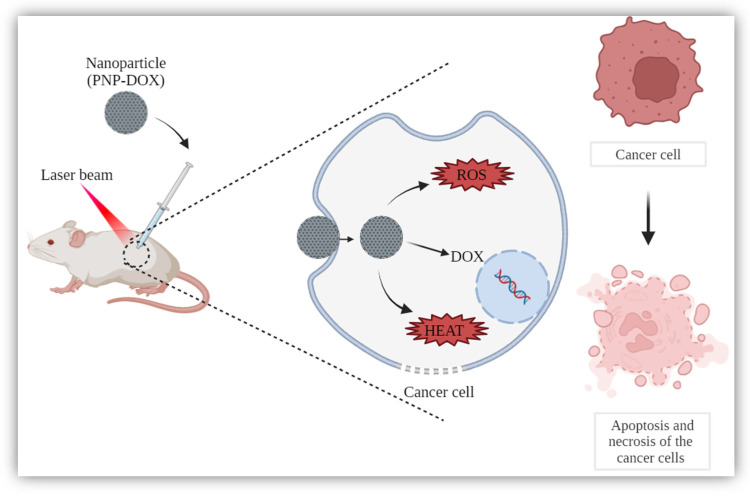
Nanotechnology in phototherapy. Created with Biorender (www.BioRender.com accessed on 9 September 2024).

The main issues of the currently used photosensitisers are insufficient selectivity, low absorption band, poor bioavailability and low efficiency. No photothermal effect is observed as well as the capability to co-deliver chemotherapeutic drugs. In response to these limitations, Lin et al. (2016) developed a multifunction nanoporphyrin platform (PNP), which was surrounded by the specific ligand PLZ4. Through the combination of a porphyrin–cholic acid (CA) conjugate, PEG and cholic acid conjugate with polyethylene glycol, it was possible to receive a nanoplatform. This enabled the simultaneous execution of photodynamic diagnosis, image-guided photodynamic therapy, photothermal therapy, and targeted chemotherapy [97]. The literature indicates that PLZ4 specifically binds to the αvβ3 integrin on to canine invasive transitional cell carcinoma (TCC) cell lines (K9TCC-PU, K9TCC-PU-AxA, K9TCC-PU-In, K9TCC-PU-AxC, and K9TCC-PU-Nk) [98]. PNPs were created with the use of a mixed micelle strategy. Through the solvent evaporation method, DOX was loaded. Study results showed that PNP could be specifically taken up by human bladder cancers in a time-dependent manner and distributed in the cytoplasm. The presence of the PLZ4 on the PNP surface enhanced internalisation by bladder cancer cells, which was linked to increased cytotoxicity. PNP cytotoxicity after illumination was >100 times stronger compared to 5-ALA in vitro (5637 bladder cancer cells). Light exposure to PNP-pretreated BC cells caused cellular damage, including cellular and nuclear swelling, loss of cell–cell contact and degradation of the membrane. The intravesical installation of PNP loaded with DOX (PNP-DOX) to an orthotopic PDX mouse model (BL269 mice model) showed the ability of PNPs to deliver DOX into cells and then release DOX into the nucleus (Figure 1). Moreover, the anatomy of PNP-treated bladder was grossly normal. BC developed in 80% of orthotopic PDX BC models treated with PBS and free-DOX, while minor tumours occurred only in 25% of the PNP group. After the application of light (90 J/cm^2^) in the PNP-DOX group, a longer inhibition of the tumour growth was discovered also with a remission after three cycles of therapy. This demonstrates that the associated therapy greatly declined the lifespan of the cells. Most importantly, in all therapeutic groups, no declines in body weight were observed nor symptoms of systemic toxicity. In the experiment, also the PNP’s capability in the diagnosis was tested. The designed PNPs allowed the detection of minor cancerous changes in the bladder. To summarise, the use of doxorubicin (DOX)-loaded PNPs allowed slow drug release and longer systemic circulation time, simultaneously indicating selectivity only toward bladder cancer cells. Furthermore, PDT with PNPs was more effective than 5-aminolevulinic acid [97].

## 5. Application of Nanoformulations in Gene Therapy

Advancements in molecular biology allow genetic changes responsible for BC development to be identified. Many genes in BC pathogenesis were identified, and their relation to clinical outcomes was noted. It is commonly assumed that there are two pathways for BC development, which are the activation of genes that accelerate cancer growth (*RAS*, *FOS*, *MYC*, *ERBB-2*, *CCND1*, *EGF*, *EGFR*, *BCL-2*, *BIRC5*, *VEGF*, *bFGF*, *COX-2*) and suppression of the genes that slow down the proliferation of the cells (*p53*, *p21*, *PTEN*, *ARF*, *CDKN2A*, *TRAIL*, *RB*, *BAX*, *FAS*, *GSN*, *TNF*, *ICAM-1*, *CCAM-1*, *CAR*, *CDH*). The progress has allowed the creation of a new therapy for BC based on gene production [99]. Gene therapy is an innovative treatment strategy, which targets DNA or RNA to inhibit the uncontrolled growth of cancer cells or to modulate the immune system. The therapy consists of the choice of a specific gene, delivery of the genetic material to the targeted cell and ensuring that expression of the gene by the cell occurs [100]. The delivery of the gene into the cell is the most important consideration to be taken into account in BC treatment. Viral vectors were studied; however, they failed to demonstrate suitable therapeutic gene transduction. This can be related to the glycosaminoglycan (GAG) overlaying the bladder mucosa [101]. They exhibit poor target cell specificity, are unable to carry large genes, and are expensive. Another obstacle is the blood–tumour barrier (BTB), which influences the penetration of nucleic drugs into the cancer cells. Moreover, in vivo research demonstrated a high percentage of degraded blood-circulating RNA genes. To overcome this problem, some strategies have been proposed, such as improving the non-viral delivery systems and disruption of the GAG layer, including nanoparticles [99]. In addition to the efficient and safe transport of genes to the target cell, the effectiveness of gene therapy also depends on the efficient monitoring of modified cells or factors using non-invasive imaging techniques, which will enable the tracking of gene delivery and expression of modified genes. The application of nucleic acid drugs associated with nanoparticles could improve intracellular accumulation in tumour tissue and protect against enzymatic degradation [102].

**Figure 2:**
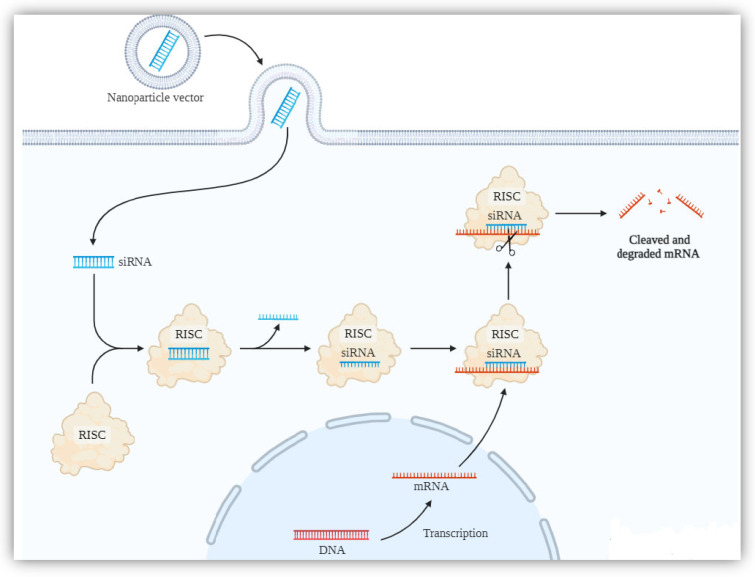
Nanotechnology in gene therapy. Created with Biorender (www.BioRender.com accessed on 9 September 2024).

One of the ways to lower gene expression during BC therapy is the application of RNA interference (RNAi). Among RNAi, siRNA and shRNA significantly suppress the proliferation and invasion of cancer cells and also reverse drug resistance (Figure 2) [103]. To address this, Liang et al. (2020) developed chitosan–hyaluronic acid dialdehyde (CS-HAD NPs) nanostructures to deliver *BCL-2*-siRNA for tumour growth inhibition. Hyaluronic acid dialdehyde (HAD) was prepared by using an ethanol–water mixture and conjugating it to amine groups of chitosan nanostructures. The obtained CS-HAD NPs (100–120 nm) were able to selectively deliver siRNA to cancer cells with overexpressed CD44. siRNA was loaded into the core of NPs with an efficiency of 95% (siRNA@CS-HAD NPs). They were characterised by high biocompatibility, stability, and selectivity against tumour cells. To determine the efficiency of the appliance system and the effectiveness of BC treatment, research was conducted both in vitro and in vivo. After the incubation of T24 cells with siRNA@CS-HAD NPs, the viability of the BC was significantly reduced, and increased levels of cleaved PARP were observed. The results showed that siRNA@CS-HAD NPs could be efficiently internalised with T24 cells through a receptor (CD44)–ligand (HA)-mediated effect and could target gene silencing. Blood compatibility evaluation indicated that the use of siRNA@CS-HAD NPs did not impact the red blood cells. To evaluate antitumour effects, nude mice received intravenous injections. The growth of tumour T24 was significantly inhibited by siRNA@CS-HAD NPs compared to naked siRNA or siRNA@CS NPs. The investigated delivery system could be proposed as a novel treatment method for BCs, which show a high expression of CD44 [104]. Some of the cancer cells also show high expression of other factors, allowing them to extend the cell’s life. Survivin is an inhibitor of apoptosis, which is expressed in the G2/M phase of the cell cycle. According to the in vitro studies, survivin can inhibit the effector cell death caspases 3 and 7. This cytoplasmic protein is present during foetal development but is generally absent in fully differentiated adult tissues (except normal human endometrium during the proliferative phase and prostatic neuroendocrine cells) [105]. Martin et al. (2014) modified biodegradable PLGA nanoparticles with chitosan (CH2.5, CH20) to deliver survivin siRNA [106]. Nanostructures were synthesised by a double-emulsion solvent evaporation technique, and the efficiency encapsulation of siRNA was 70%. The modification of NPs with chitosan caused a 10-fold increase in internalisation by bladder tumour cells when compared with unmodified NPs. The transfection of UM-UC-3 BC cells with NP-siSUR-CH2.5 or NP-siSUR-CH20 decreased the level of viability by 75% and reduced tumour volume by 65%. Interestingly, the bioactivity of survivin siRNA was extended up to 9 days and resulted in a decreased proliferation rate [106].

The ncRNAs are functional RNA that do not translate into proteins. Their main functions include the regulation of biological processes such as differentiation, proliferation and migration. MicroRNAs (miRs) [103] are a group of short endogenous, non-coding RNAs, which induce a gene-silencing effect through binding to the 3′-untranslated regions (3′-UTRs) of multiple target messenger RNAs (mRNAs). miRs can be divided into two groups based on their functions: tumour suppressor miRs and oncogenic miRs (oncomiRs) [107]. The faulty expression of particular miRs is connected with the pathological events of most solid tumours [103,107]. MiR-34a acts as a tumour suppressor and downregulates most human malignancies. Altered expressions are related to cell proliferation, angiogenesis, migration, and metastasis. The use of miR-34a in target therapy could downregulate the expression of C44 in cancer cells as well as enhance the sensitivity of the tumour cells to chemotherapeutic agents. Shahidi et al. (2022) constructed biocompatible, modified mesoporous silica nanoparticles (c(RGDfK)-MSN NPs) for the co-delivery of miR-34a and siPD-L1 to BC cells [107]. PD-L1, also called programmed death-ligand 1, is a transmembrane protein that can act as a biomarker to predict response to chemotherapy and clinical outcomes among MIBC patients who underwent radical cystectomy. PD-L1 is located on most cancer cell surfaces. Its main role in pathogenesis is blocking T-cells through binding to its receptor (PD-1) and protecting cancer cells from T-cells. Therefore, by using PD-1/-PD-L1 inhibitors, it is possible to obtain remarkable outcomes in BC therapy. Simultaneously, the delivery of the mentioned particles (miR and siPD-L1) will downgrade expression in both PD-L1 and CD44 and increase apoptosis through the sensitisation of cancer cells. In this study, various techniques were used to determine in vitro and in vivo anticancer activities. Their nanoformulation required blood stability for cancer therapy. The release of miR-34a and siPD-L1 from c(RGDfK)-MSN NPs was pH-dependent, and a significant “launch” of small RNAs was observed at acidic pH (5.4). The designed c(RGDfK)-MSN NPs were successfully internalised by T24 cells and had no toxic effects. An in vitro study on T24 cells showed lower expression levels of siPD-L1 and higher levels of miR-34a in the c(RGDfK)-MSN NPs-treated group. Notably, migration and invasion within T24 cells were reduced to 55% and 45%, respectively. In vivo research on nude mice c(RGDfK)-MSN NPs had high tumour selectivity and the ability to inhibit tumour growth [107].

## 6. Application of Nanoformulations in Immunotherapy

Immunotherapy is an innovative method of treating oncological patients who are undergoing intensive studies. In the past few years, the FDA approved a significant number of immunological drugs, while many forms of therapy are in the clinical and pre-clinical phases. Immunotherapy, in contrast to chemotherapy, focuses on modulating the anticancer immune response and targeting the natural “artillery” of the immune system against cancer cells [108,109]. Unlike other genitourinary cancers (for example prostate cancer), BC is characterised by an increased number of immune gene expression patterns and enhanced immune checkpoint genes in tumours. Therefore, it is an ideal candidate for immunotherapy [110]. Although immunotherapy has the potential to improve outcomes in patients with advanced urologic malignancies, it is still associated with systemic immune-related adverse events (irAEs) and poor targeting due to the complex tumour microenvironment. The solution to this problem may be to combine immunotherapy with nanoparticles. As mentioned, nanoparticles are small in size, which results in high transport efficiency [111] to specific sites or cells. Nanoparticles act precisely on tissues or cells, enhancing the immune response and reducing irAE [112]. Moreover, nanoparticles as transporters can carry two or more drugs to enhance synergistic effects [113]. Importantly, nanoparticles can be used to recognise not only cancer cells but also matrix metalloproteinases (MMPs) or lysoxidase (LOX) in the tumour microenvironment (TME), thus realising dual recognition. Furthermore, the use of nanoparticles can induce immunogenic cell death (ICD) and release danger-associated molecular patterns (DAMPs) to promote dendritic cell (DC) maturation, and additionally, nanoparticles can also transform M2-type macrophages into M1-type macrophages in the TME. Importantly, immunotherapeutic agents coupled to nanoparticles can be delivered to tumours in a much shorter time while clearing them from the body’s circulation more rapidly, providing similar stimulation in the TME but significantly reducing systemic exposure [111].

One of the applications of nanoparticles is therapy with the BCG vaccine. During treatment with BCG, a partial reaction and a systemic reaction can occur, leading to life-threatening sepsis. For this reason, efforts were made to find a focused system for BCG applications [103]. Erdoğar et al. (2015) used cationic chitosan nanoparticles for the safe and effective intravesical delivery of BCG. CS nanoparticles were synthesised using an ionotropic gelation technique, and the EE% was 42%. The obtained BCG nanoformulations were characterised by high biocompatibility and non-toxicity. The intravesical administration of BCG nanosolution led to increased antitumour efficacy, increased survival rate, and reduced side effects [114]. BCG-CWS (BCG cell wall skeleton) can be the alternative to live BCG. However, because of its poor solubility and strong negative charge, clinical application for cancer treatment is limited. To overcome this problem, Nakamura et al. (2014) encapsulated BCG-CWS into lipid nanoparticles [115]. CWS-NPs were prepared by liposome evaporated via the emulsified lipid (LEEL) method. Here, 95% of MBT-2 cells had taken up constructed CWS-NP/LEEL. Moreover, among the mice with BC, treatment with CWS-NP/LEEL significantly suppressed tumours. Intravesical administered CWS-NP/LEEL in a rat model decreased the tumour’s size, and it was dose-dependent. To determine clinical applications, CWS-NP/LEEL studies have been conducted on human Th1/Th2 cell differentiation. The results showed an increased level of IFN-γ-producing cells (Th1 culture) and a lowered level of IL-4-producing cells (Th2 culture). Due to the potential of the described solution, it is important to note that this study should be broadened by studies of non-clinical, clinical and GMP control [115].

Since 2016, the FDA has approved five immunological control point inhibitors (ICIs) in MIBC therapy. ICIs are a part of monoclonal antibodies directed against cytotoxic T-lymphocyte-associated antigen 4 (CTLA-4), programmed death 1 (PD-1) receptor and programmed death ligand-1 (PD-L1) [116,117]. Among ICIs, we can distinguish atezolizumab, pembrolizumab, avelumab, durvalumab, and niwolumab [117,118,119,120]. A major obstacle to cancer immunotherapy is the controlled modulation of the immune system. Among patients subjected to such therapy, the occurrence of side effects such as autoimmunity and non-specific inflammation has been documented. Therefore, understanding the way to increase the response rates to various classes of immunotherapy would allow the improvement of efficacy and control of adverse effects. The use of nanoparticles as a delivery system could be a solution to all the above-mentioned problems [108]. In 2021, Zhou et al. conducted research to effectively apply multiple drugs to the tumour tissue simultaneously [116]. The main goal of applying combined immunotherapy for BC is the abolition of the inhibitory effect of the tumour microenvironment on immune effector cells and improving the efficacy of immune checkpoint inhibitors. Therefore, macrophage-derived exosome-mimetic nanovesicles (EMVs) were constructed to deliver the CD73 inhibitor (AB680) and the monoclonal antibody (aPDL1). Based on the in vitro and in vivo studies, a high biosafety profile, good stability, and targeted delivery of AB680@EMVs-aPDL1 to BC was determined. Interestingly, CD73 reduced the generation of extracellular adenosine, while PD-L1 downregulation prevented immune escape. The investigated combination therapy caused the increased activation and infiltration of cytotoxic T-lymphocytes, leading to an inhibition of tumour growth and extended survival in mouse BC models [116]. Li et al. (2022) used the combination of immune-stimulating agent interleukin-12 and chemotherapeutic epirubicin (THP) for the elimination of orthotopic bladder tumours and lowering the chance of remission. Synthesised fluorinated chitosan (PGFCS) could open tight junctions in bladder epithelium, which enhanced penetration into the tumour site. The intravesical application of nanoformulated THP and IL-12 caused effective antitumour action [121].

## 7. Conclusions, Challenges, and Future Prospects

The treatment of BC is a complex problem for modern oncology. Current BC treatment methods include radical and non-bladder tissue-specific approaches, such as RC. Currently, the transurethral resection of non-muscle-invasive bladder cancer followed by intravesical chemotherapy is the gold standard treatment approach to minimise recurrence and delay the progression of cancer. Unfortunately, the effectiveness of this therapy is not satisfactory. This is a consequence of several factors: (a) the activity of drugs is not limited to cancer cells, which results in a limited killing effect on tumour cells and contributes to significant toxicity to the normal bladder mucosa; (b) due to continuous urine production, the concentration of intravesical drug in bladder cancer tissue decreases with increasing urine volume in the bladder, and in addition, continuous urine output contributes to a shortening of its retention time in the bladder; (c) due to limited retention, the drug is administered frequently, which in turn affects severe local irritation symptoms; (d) the efficacy of intravenous drug is limited by the biological barrier of the bladder epithelium [122].

Therefore, in recent years, many attempts have been made to modify conventional anticancer therapy, minimising previous shortcomings by combining innovative technology with the use of various nanoparticles. Previous studies have confirmed the great potential of using nanoparticles in both the diagnosis and treatment of various cancers, including improved selectivity and sensitivity in the treatment of bladder cancer [16]. The undoubted advantage of nanoparticles used in medicine is their ability to bypass the side effects typical of conventional therapy by improving the specificity and pharmacokinetics of traditional anticancer drugs. Nanomaterials are characterised by their large specific surface area, strong adsorption capacity, high bioavailability, precise targeting features and controlled drug release rate. Moreover, the use of nanoparticles is aimed at improving stability and increasing the affinity of transported drugs, promoting specificity and targeting therapy to cancer cells [16,122].

Among the wide range of available nanoparticles, polymeric nanoparticles, including chitosan, are worth mentioning, as their synthesis is easy and cost-effective and easily biodegradable. As already mentioned, chitosan is a non-toxic and biocompatible polymer that contributes to the better penetration of anticancer drugs into the deeper layers of bladder cells by temporarily eliminating the barrier function of the bladder epithelium. The undoubted advantage of chitosan is its electrostatic interactions with mucins in the mucus layer, which contributes to increased bioadhesion, making chitosan an ideal polymer for intravesical administration. Moreover, chitosan can also promote the structural reorganisation of tight junction proteins, resulting in a better absorption of hydrophilic drugs. These properties make chitosan an excellent candidate for intravesical drug delivery. However, there is still a paucity of studies on the biodistribution and pharmacokinetics of chitosan. Moreover, the in vivo studies conducted to date are focused on rat and mouse animal models, and therefore, they need to be expanded to include studies on large animal models to determine whether these new drugs will be clinically acceptable. Nevertheless, chitosan has high clinical potential [123,124,125,126,127].

The dynamic development of nanomedicine has also contributed to the development of various types of lipid-based nanoparticles. So far, it has been studied in BC therapy using (a) liposomes, (b) lipid nanoemulsions, (c) lipid nanoparticles, (d) solid lipid nanoparticles, and (e) nanostructured lipid carriers. The advantages and disadvantages of the individual types of lipid-based nanoparticles are presented in Table 7 [126,127,128,129].

The activity of lipid-based nanoparticles is mainly based on the EPR effect. The abnormal vascularisation and impaired lymphatic drainage often observed in tumour tissues are referred to as the EPR effect. Tumours often have leaky blood arteries with irregular fenestrations, allowing passive nanoparticles to escape from the bloodstream into the tumour interstitium. At the same time, impaired lymphatic drainage from tumours impedes the effective removal of these nanoparticles. On the other hand, the selective accumulation of therapeutic-bearing nanoparticles can be increased through the surface modification of the nanoparticles with tumour cell-specific ligands that can recognise and bind to complementary molecules or receptors overexpressed on the surface of the targeted cells. Active targeted transport provided by lipid-based nanoparticles further reduces systemic side effects by enhancing drug delivery to tumour cells while minimising the exposure of normal tissues to the toxic therapeutic agent [130].

Importantly, lipid-based nanoparticles can be used to transport both lipophilic and hydrophilic compounds. Interestingly, due to their exceptional biocompatibility, biodegradability, and entrapment efficiency, lipid-based nanoparticles seem to be suitable as carriers for nucleic acids such as DNA, mRNA, and siRNA [131].

The undoubted advantage of lipid-based nanoparticles is both biocompatibility and biodegradability. In addition, the undeniable advantage of using these nanoparticles as drug carrier systems is that the matrix consists of physiological components, i.e., excipients with GRAS status for oral, local, and intravenous administration, which significantly reduces possible cytotoxicity. Lipid-based nanocarriers protect labile drugs from degradation or rapid clearance, which is particularly important for short-lived drugs used in anticancer or gene therapy. The undoubted advantage of lipid-based nanoparticles is the possibility of their oral administration instead of the less comfortable intravenous route for the patient. Previous studies suggest that lipid nanoparticles are absorbed by the lymphatic system after oral administration. This allows for achieving high drug concentrations in lymph nodes, which may be crucial in the therapy of metastatic cancers and the treatment of lymphoma. However, further studies are necessary to fully characterise the lymphatic absorption of lipid nanoparticles [132].

The next nanoparticle with clinical potential in the treatment of BC analysed in this article was protein nanoparticles. Compared to other nanosystems used in BC therapy, protein-based nanoparticles have several unique advantages. (1) They are highly biocompatible, biodegradable, and have low immunogenicity. (2) They are easy to manufacture—they can be produced without the use of chemical synthesis and toxic solvents [133]. (3) The surface of protein nanoparticles can be easily modified to improve their function due to the presence of numerous functional groups on their surface [134]. (4) The protein is naturally amphipathic, which allows the hydrophobic domain of proteins to interact with many hydrophobic anticancer drugs to improve the drug encapsulation ability of the nanoparticles. (5) Some proteins have a natural tropism for tumours, and other targeting ligands can also be modified on the surface of protein nanoparticles to target specific tumours [135]. (6) Due to the non-toxicity and high drug encapsulation capacity, protein-based nanoparticles used for loading drug cargo can achieve higher drug concentrations inside the tumour [136]. (7) The hollow structure of some proteins enables the convenient loading of small drug molecules or metal nanoparticles for drug delivery and combination therapy. (8) The metabolic products of protein nanoparticles are amino acids, which are non-toxic and harmless to humans. However, there are some significant limitations in the clinical application of protein nanoparticles. Some proteins that can potentially be used as anticancer drug transporters are not so stable, and their structure may change during the synthesis of nanoparticles. Moreover, we still do not have sufficient knowledge about the transport mechanism via protein nanoparticles. The studies on the circulation time, biodistribution and mass production of protein nanoparticles are very limited. Moreover, protein nanoparticles also face the problem of low drug loading rate. On the other hand, nanoparticles smaller than 20 nm or larger than 250 nm can be easily cleared by the kidneys or reticuloendothelial system, which in turn affects the drug circulation time, reducing the efficacy of the treatment [137].

The advantages and disadvantages of the described nanoparticles are summarised in Table 8.

To sum up, there is currently an increased interest in nanotechnology and a related rapid increase in the number of studies on various nanoparticles. However, only a few developed nanoparticles reach the clinical trial phase. Most of them are halted at the in vivo and in vitro studies stage. The challenges for nanoparticles include the lack of routes of administration, inhibition of biodistribution, the channel of NPs across the biological barriers, their degradation and toxicity. Nanoparticles are usually administered intravenously, which limits their transport against blood flow, retention and interaction with the target site, which consequently may not provide the desired therapeutic effects [138]. A solution to this problem may be magnetic nanoparticles, as in vivo and in vitro studies have shown their ability to move in a controlled manner under the influence of a magnetic field against blood flow. However, the effect of magnetic fields on the human body used to change the flow direction of these nanoparticles has not yet been assessed.

Another problem is the detailed understanding of the biological fate of nanoparticles in the body—metabolism, biodistribution, and clearance. Unfortunately, controlling the biological fate of NPs is very difficult. Although nanoparticles are made of biosafety materials and are appropriately modulated to increase the retention time and half-life, there is a risk of damage to the lungs, liver, and kidneys. Nanoparticles accumulating in the lungs exhibit inflammatory, oxidative, and cytotoxic effects [139]. Moreover, nanoparticles induce the formation of free radicals, which have a toxic effect on normal cells [140]. This problem is being solved by modulating the surface, size, and shape of particles and their solubility [141] as well as manufacturing nanoparticles from more biocompatible substances, such as chitosan or materials that disintegrate after irradiation with near-infrared light.

An important problem of nanomedicine is also related to the interactions of nanoparticles with the mononuclear phagocytic system. Once nanoparticles enter the body, they interact with the host immune system and are engulfed by cells of the mononuclear phagocyte system (MPS). This interaction can lead to immunosuppression or immunostimulation, which in turn can enhance or attenuate the effects of nanoparticle treatment. Currently, commonly used strategies are preventing these processes, depleting and reprogramming TAMs (tumour-associated macrophages), and blocking “CD47–SIRPα pathways” [142].

The major technological challenges associated with nanoparticle therapy are scale-up synthesis, uniform optimisation, and yield prediction. These are key to securing the clinical success of nanoparticle therapy. Most nanoparticles used in in vivo and in vitro studies are usually produced in small batches, and scaling up to huge quantities is not always feasible due to technical reasons, e.g., lack of availability of appropriate instruments. Therefore, replicating the manufacturing success on a large scale is a significant challenge in terms of economics, organisation, and calibration. Comprehensive studies using computational and theoretical modelling combined with experimental studies can help solve this problem [143].

Clinical application is also limited by the design of clinical trials. Despite positive results of in vitro and in vivo studies, it is difficult to predict how to design therapy for patients—for example, how long the therapy will last. Additionally, therapies using nanoparticles are usually second-line therapies. This means that nanopreparations will be administered to people who have acquired drug resistance as a result of previous therapies as well as to those with tumour progression and metastases. Such situations often distort the results of clinical trials and reduce the chance that nanoparticle therapy will deliver benefits to those who are likely to respond to the proposed therapy, i.e., patients with primary tumours. Moreover, in case of nanomedicine, the optimisation of personalised therapy seems to be extremely difficult. Planning such therapy requires the assessment of the influence of many factors, including genetics, environment and previous medical history. Such clinical trials are associated with exorbitant costs and are time consuming because the optimisation of such therapy requires treating each patient qualified for treatment as a separate clinical trial (N = 1) [143].

In summary, nanoparticles show high therapeutic potential, especially in oncology. However, it should be remembered that before introducing nanopreparations to the market, several studies must be conducted to ensure both the effectiveness and safety of nanotherapy.

## Figures and Tables

**Table 1:** BC stages.

Tumour Stage	Description
CIS or Tis	The inner lining layer of the bladder
Ta (*NMIBC*)	Non-invasive papillary carcinoma
T1 (*NMIBC*)	Invades lamina propria
T2 (*MIBC*)	Invades muscularis propria
T3 (*MIBC*)	Invades perivesical tissue
T4 (*MIBC*)	Extravesical extension into adjacent organs

**Table 2:** Nanomedicines for BC in clinical trials. Based on [43].

Drug/Drugs	Nanocarriers	Disease	Sponsor/Collaborators	Status	NCT Number
EP0057	Polymeric	Urothelial Carcinoma, Urothelial Cancer, Lung Neoplasms, Small Cell Lung Cancer, Prostate Cancer	National Cancer Institute (NCI)	Recruiting	NCT02769962
Imx-110	Polymeric	Solid Tumours	Immix Biopharma Australia Pty Ltd. (Los Angeles, CA, USA)	Active, but not recruiting	NCT03382340
PLZ4-coated paclitaxel-loaded micelles (PPM)	Micelles	NMIBC	VA Office of Research and Development	Recruiting	NCT05519241

**Table 4:** PNPs in BC drug delivery.

Nanocarrier	Drug	Amount of Nanoparticle Formulations	Results	Study Type	Days of Study	Ref.
Poly (L-aspartic acid sodium salt; PAA) with or without different densities of PEG (high or low)	Cisplatin (CP)	0.7 mg/mL in 100 μL or 300 μL	Decreased local toxicity, reduced systemic exposure, sustained release of CP and high antiproliferative activity.	In vitro (superficial bladder cancer cell line (RT4), invasive cell lines (J82, 5673)/In vivo (female CF-1 mice)	21 days	[71]
Amphiphilic poly(ε-caprolactone)-b-poly(propargyl methacrylate-click-mercapto succinic acid-co-poly(ethylene glycol) methyl ether methacrylate)	Cisplatin (CP)	10 μM	Sustained release of CP and high antitumour activity.	In vitro (human urothelial carcinoma cell line UMUC3)	-	[72]
Hyperbranched polyglycerols (HPGs) modified with MePEG and amine groups (HPG-C8/10-MePEG-NH2)	Docetaxel (DTX)	0.2 mg/mL in 50 μL	High antitumour activity and enhanced uptake by bladder tumour tissues.	In vivo (female nude mice)/In vitro (low-grade human urothelial carcinoma cell (RT4, MGHU3), high-grade human urothelial carcinoma cell (UMUC3)	25 days	[74]
Oligoarginine-poly(ethylene glycol)–poly(L-phenylalanine-co-L-cystine) (R9-PEG–P(LP-co-LC))	10-Hydroxycamptothecin (HCPT)	6.0 mg/kg	High antitumour activity and low systematic toxicity.	In vitro (human BC 5637)/In vivo (C57Bl/6 mice, male SD rats)	35 days	[75]

**Table 5:** Lipid-NPs in BC drug delivery.

Nanocarrier	Drug	Amount of Nanoparticle Formulations	Results	Study Type	Days of Study	Ref.
Maleimide-functionalised PEGylated liposomes (PEG-Mal)	NaFI (model drug)	20 μL/100 μL/2 mL	High penetrating ability, retention and drug release profiles.	In vitro (porcine urinary bladder tissues)	-	[86]
Soybean phosphatidylcholine (SPC) and hydrogenated SPC (HSPC)	Curcumin	N/A	Sustained release of curcumin, increased cellular uptake and cytotoxicity.	In vitro (bladder carcinoma HTB9 cell line, normal fibroblast L929 cell line)	-	[88]
Nanomicelles	Curcumin	80 mg	Good toleration, no significant difference between study and control group.	Double-blind placebo-controlled trial	28 days	[61]

**Table 6:** Protein-NPs in BC drug delivery.

Nanocarrier	Drug	Amount of Nanoparticle Formulations	Results	Study Type	Days of Study	Ref.
Gelatin nanoparticles	Paclitaxel (PTX)	1 mg/20 ml	Decreased local toxicity, sustained release, retention and high concentration of PTX.	In vitro (phosphate-buffered saline (PBS)/In vivo (tumour-free dogs and tumour dogs)	21 days	[93]
Human albumin-nanocarrier (txCD47-HNP)	Gemcitabine elucidates (GEM)	N/A	High antitumour activity and enhanced uptake by bladder tumour tissues.	In vitro (bladder cancer (BC) cells)/In vivo (mouse orthotopic BC model)	-	[95]

**Table 7:** Characteristics of lipid-based nanoparticles based on [126,127,128,129].

Lipid-Based Nanoparticles	Advantages	Disadvantages
Liposomes	increase in efficacy and therapeutic drug index increase in drug stability thanks to the encapsulation the ability to transport both lipophilic and hydrophilic drugs non-toxic, flexible, biocompatible, biodegradable and non-immunogenic reduction in toxicity of the encapsulation drug site avoidance effect improved pharmacokinetics effects	low solubility short half-life fewer stables high production cost leakage and fusion of encapsulated drug possibility of phospholipid oxidation limited accumulation and penetration into the interstitial space of the tumour contributing to limited therapeutic efficacy
Lipid nanoemulsions	increase in efficacy and therapeutic drug index increase in drug stability thanks to the encapsulation improves pharmacokinetics increases drug solubility enhances bioavailability enables targeted drug delivery low cost production higher loading capacity controlled drug release	nanoemulsions have limitations like stability, temperature, and pH problematic for compounds with high melting points because of their poor solubility skin irritability less permeability and bioavailability of drug
Solid lipid nanoparticles	increase in efficacy and therapeutic drug index increase in drug stability thanks to the encapsulation preventing the use of organic solvents during production increase in efficacy and therapeutic drug index no toxicity because the lipids utilised are biocompatible and biodegradable materials increase in drug stability thanks to the encapsulation easy production scalability high physical stability solid lipid nanoparticles are sterilisable lipophilic and hydrophilic medications may be encapsulated	drug sensitivity to high pressure minimal drug loading potential poor kinetics of the therapeutic delivery process lipid dispersions have high water content limited transdermal medication delivery increase in particle size while being stored lipid dispersion gelation the toxicity of lipid nanoparticles on retinal cells has not yet been thoroughly investigated
Nanostructured lipid carriers	increase in efficacy and therapeutic drug index increase in drug stability thanks to the encapsulation it is non-immunogenicity, biocompatibility, biodegradability, prolonged and controlled release, large surface area and thermodynamic stability the use of generally recognised as safe (GRAS) materials, the large-scalable production, and improved drug safety allow nanostructured lipid carriers to be an attractive delivery system candidate for the pharmaceutical market drug loading potential higher than in the case of solid lipid nanoparticles may be administered orally if the formula contains biocompatible surfactants	high cost of production and formulation of nanoemulsions compared to conventional lipid nanoparticles achieving the desired properties and stability of nanoemulsion formulations can be complicated and require specialised knowledge nanoemulsions rely heavily on surfactants, and the choice of surfactant is critical. Some surfactants may have limitations or undesirable effects maintaining long-term stability can be difficult, and nanoemulsions may require additional measures to prevent destabilisation over time transitioning from laboratory to industrial production can be challenging, and this change can impact the stability and properties of the nanoemulsion

**Table 8:** Advantages and disadvantages of nanoparticles.

	Chitosan	Polymeric	Lipid-Based	Protein
Advantages	Biocompatibility and degradability Mucoadhesivity Easily enzymatically soluble Low toxicity	High encapsulation efficiency High intracellular uptake High stability of active substance encapsulation, Biocompatibility with tissues and cells	High biocompatibility High degradability Low toxicity	Highly biocompatible Non-toxic Biodegradable Non-antigenic
Disadvantages	Poor solubility at physiological pH	No biodegradability Frangibleness High manufacturing costs Toxic solvent residuals	High price	Low mechanical strength
